# Complete genome sequence of *Actinomycetaceae* bacterium strain MB13-C1-2, isolated from the feces of the black soldier fly larvae

**DOI:** 10.1128/mra.00640-26

**Published:** 2026-08-05

**Authors:** Xi Chen, Mengyan Han, Jingjing Zhao, Chih-Hung Wu, Guowen Dong, Wangchuan Xiao, Chao-Jen Shih, Yen-Chi Wu, Song Wang, Wanling Qiu, Lintao Wu, Sheng-Chung Chen

**Affiliations:** 1 School of Resources and Chemical Engineering, Sanming University 66283, Sanming City, Fujian, People’s Republic of China; 2 Fujian Provincial Key Laboratory of Resources and Environmental Monitoring and Sustainable Management and Utilization, Sanming University 66283, Sanming, Fujian, People’s Republic of China; 3 School of Chemistry and Materials, Fujian Normal University 12425, Fuzhou, Fujian, People’s Republic of China; 4 College of Environment and Safety Engineering, Fuzhou University 12423, Fuzhou, Fujian, People’s Republic of China; 5 School of Resources and Environment, Fujian Agriculture and Forestry University, Fuzhou, Fujian, People’s Republic of China; 6 Bioresource Collection and Research Center, Food Industry Research and Development Institute, Hsinchu, Taiwan, Republic of China; Indiana University Bloomington, Bloomington, Indiana, USA

**Keywords:** *Actinomycetaceae*, black soldier fly, anaerobes

## Abstract

Here, we report the complete genome sequence of *Actinomycetaceae* bacterium strain MB13-C1-2, isolated from the feces of black soldier fly (*Hermetia illucens*) larvae, comprising 2,578,609 bp and a GC content of 57.23%. The genome was selected for further analyses of species delineation and genomic features.

## ANNOUNCEMENT

**Fig 1:**
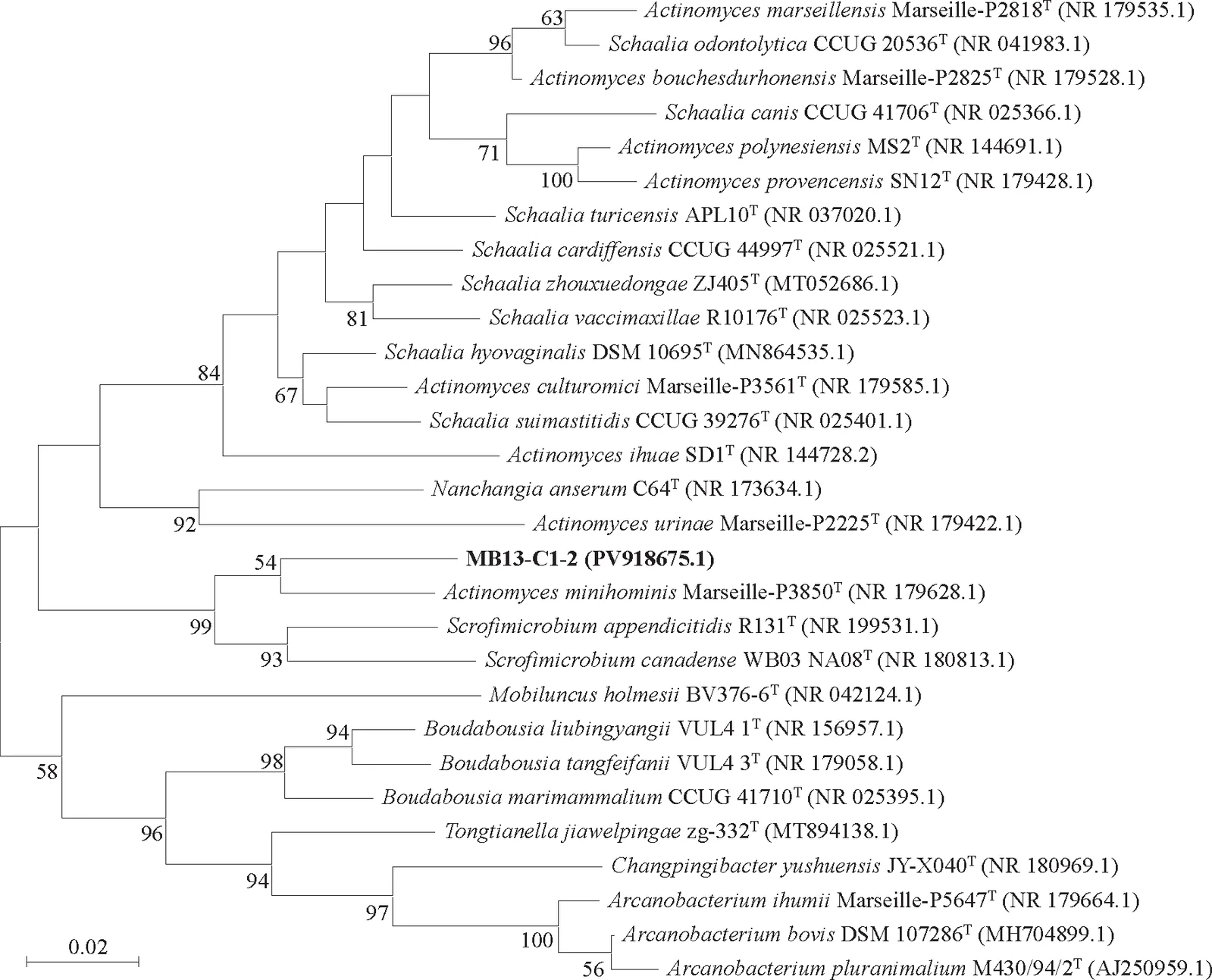
Maximum-likelihood tree based on 16S rRNA gene sequences showing the preliminary phylogenetic placement of strain MB13-C1-2 within the family *Actinomycetaceae*. Sequence alignment was performed using Clustal W (9), and the Tamura-Nei model (10) was applied as the nucleotide substitution model. Bar, 0.02 substitutions per nucleotide position. Bootstrap values were expressed as percentages of 1,000 replicates.

Strain MB13-C1-2 was isolated from the feces of *Hermetia illucens* larvae, commonly known as black soldier fly (BSF) larvae, which are widely used in organic waste recycling and as a protein source (1). BSF eggs purchased from Taobao in June 2020 were reared at approximately 25°C for several generations. For enrichment of anaerobic bacteria, the feces of BSF larvae were collected on May 30, 2021, using a sterile spoon and inoculated into MB/W medium (per liter: 1 g MgCl_2_·7H_2_O, 0.5 g KCl, 0.1 g CaCl_2_·2H_2_O, 0.4 g K_2_HPO_4_, 1 g NH_4_Cl, 10 mL trace element solution, 2 g yeast extract, 2 g tryptone, 0.5 mL Na-resazurin solution (0.1%), 4 g NaHCO_3_, vitamin solution, 0.25 g L-cysteine-HCl·H_2_O, 0.25 g Na_2_S·9H_2_O, headspace: N_2_:CO_2_ = 4:1) (2, 3) and incubated at 25°C for 1 month. The strain was isolated by serial dilution and the rolling-tube technique (4) using MB/W medium at 25°C. The isolate obtained from one of the picked colonies was identified by 16S rRNA gene clone sequencing using primer pair 8F/1492RU (5). Culture purity was verified by morphological observation, 16S rRNA gene analysis, and genome sequencing. Based on BLASTN analysis (6), strain MB13-C1-2 (PV918675.1) showed the highest similarity of 94.27% to *Actinomyces minihominis* Marseille-P3850^T^ (7). The 16S rRNA gene-based phylogenetic tree constructed using MEGA11 (8) provided a preliminary placement of strain MB13-C1-2 within the family *Actinomycetaceae* (Fig. 1).

Strain MB13-C1-2 was deposited in the Biological Resource Center, National Institute of Technology and Evaluation (NITE), Japan, under the accession number NBRC 117461. The strain was cultivated in MB/W medium at 30°C, and genomic DNA was extracted using a modified method based on Johnson (11) and Jarrell et al. (12). Briefly, cells harvested from a 500 mL culture were lysed with 1% SDS, and genomic DNA was purified by phenol-chloroform extraction and ethanol precipitation. DNA concentration and quality were assessed by UV-Vis spectrophotometry.

Whole-genome sequencing was performed using Illumina Novaseq 6000 platform and Oxford Nanopore MinION (Guangdong Magigene). For Illumina sequencing, libraries were prepared with the ALFA-SEQ DNA Library Prep Kit (FINDROP, Guangzhou), assessed by Qubit 4.0 Fluorometer (Life Technologies) and Qsep400 (Houze Biological Technology), and sequenced to produce 150 bp paired-end reads. Raw reads were processed using fastp v0.23.4 (13) with default parameters, including automatic paired-end adapter detection and trimming, a qualified-base Phred score threshold of 15, a maximum proportion of 40% low-quality bases per read, and a minimum read length of 15 bp. After quality control, 11,435,356 clean reads were retained (Table 1).

**TABLE 1:** Genome characteristics of *Actinomycetaceae* bacterium strain MB13-C1-2

Characteristic	Value
Sequencing and assembly features	
No. of Illumina clean reads	11,435,356
No. of Nanopore clean reads	360,944
Total no. of Illumina bases	1,711,600,747 bp
Total no. of Nanopore bases	1,826,566,722 bp
Genome coverage (Illumina, Nanopore)	671×, 708×
Genome features	
Chromosome size (GC content)	2,578,609 bp (57.23%)
Total no. of genes	2,303
No. of coding sequences	2,247
No. of pseudogenes	22
No. of rRNAs	6
No. of tRNAs	47
No. of noncoding RNAs	3

For MinION sequencing, genomic DNA was not mechanically sheared but was size-selected using a BluePippin system (Sage Science) with a lower cutoff of 10 kb. The sequencing library was prepared using the SQK-LSK109 Ligation Sequencing Kit and sequenced on an R9.4.1 flow cell. Base calling used Guppy v6.5.7 (high-accuracy model), and reads were filtered with NanoFilt v2.8.0 (14), yielding 360,944 reads (*N*_50_ = 10,350 bp; average = 5,060.32 bp; total = 1,826,566,722 bp; Table 1).

Hybrid *de novo* assembly was performed with Unicycler v0.5.0 (15), which identified and trimmed overlapping ends to produce a circular chromosome. The assembled chromosome was not rotated to a specific starting gene or genomic position. The complete genome of strain MB13-C1-2 was 2,578,609 bp in length and consisted of a single circular chromosome with a G + C content of 57.23% (Table 1). The average short-read and long-read coverages were 671× and 708×, respectively. Genome annotation was performed upon submission to NCBI using the Prokaryotic Genome Annotation Pipeline (PGAP) v6.6 (16). Default parameters were used for all software tools unless otherwise stated.

## Data Availability

Both Illumina and Nanopore raw reads have been deposited in NCBI and are available through the SRA database, accession numbers SRR38785544 and SRR38785543, respectively. Assemblies have been deposited in GenBank under the accession number CP141731.1. The version of the genome described in this paper is the first version. The BioProject and BioSample accession numbers are PRJNA1054007 and SAMN38879875, respectively.
